# Supplementary material for: Sociality and interaction envelope organize visual action representations
Source: Nat Commun. 2020 Jun 12;11:3002. doi: 10.1038/s41467-020-16846-w (PMC7293348; doi:10.1038/s41467-020-16846-w)
Supplement: Supplementary file 1 — Supplementary Information [file 41467_2020_16846_MOESM1_ESM.pdf]

Supplementary Information

"Sociality and Interaction Envelope Organize Visual Action Representations"

Tarhan & Konkle (2020)

SUPPLEMENTARY METHODS

fMRI Data Acquisition. Imaging data were collected using a 32-channel phased-array head coil with a 3T Siemens Prisma fMRI Scanner at the Harvard Center for Brain Sciences. High-resolution T_1-weighted anatomical scans were acquired using a 3D MPRAGE protocol (176 sagittal slices; FoV = 256 mm; 1x1x1 mm voxel resolution; gap thickness = 0 mm; TR = 2530 ms; TE = 1.69 ms; flip angle = 7 degrees). Blood oxygenation level-dependent (BOLD) contrast functional scans were obtained using a gradient echo-planar T_2* sequence (84 oblique axial slices acquired at a 25° angle off of the anterior commissure-posterior commissure line; FoV = 204 mm; 1.5x1.5x1.5 mm voxel resolution; gap thickness = 0 mm, TR = 2000 ms; TE = 30 ms; flip angle = 80 degrees; multi-band acceleration factor = 3).

fMRI Analysis and Pre-Processing. Functional data were pre-processed using Brain Voyager QX software version 2.8.4 (Brain Innovation, Maastricht, Netherlands). Functional preprocessing included slice scan-time correction, 3D motion correction, linear trend removal, temporal high-pass filtering (0.008 Hz cutoff), spatial smoothing (4 mm FWHM Kernel), and a transformation to Talairach coordinates. Whole-brain random-effect group GLMs were fit separately for each video set, as well as for both odd and even runs of each video set. In all cases, the design matrix included regressors for each condition block, specified as a square-wave regressor for each 5-second stimulus presentation time, convolved with a 2-gamma function that approximated the idealized hemodynamic response. Across these GLMs, the average variance inflation factor across conditions of the design matrix was 1.03 (where a value greater than 5 is considered problematic), and the average efficiency was 0.2[1]. Voxel time series were normalized within a run using a z-transform and corrected for temporal autocorrelations during GLM fitting. Beta weights extracted from these group-level random-effects GLMs were averaged across subjects for each voxel, and then taken as the primary measure of interest for all subsequent analyses. Each subject's cortical surface was reconstructed from the high-resolution T_1-weighted anatomical scan using Freesurfer software, and one subject was selected as the display brain for the group data.

fMRI Experiment Design Details. Participants completed 8 functional runs of the experiment. Each video set was presented in four separate 6.2-minute runs. During each run, participants saw all 60 videos from one of the two sets. Each 2.5-second video was presented twice in a row, fading in and out of a uniform gray background over a 500-millisecond time window at the onset and offset of each presentation to prevent visually-jarring transients between video presentations. Thus, each video was presented in a 5-second block. In addition, four 15-second blocks of fixation were interspersed throughout the run, placed so that no fixation blocks occurred within five blocks of each other or the beginning or end of the run. In addition, fixation periods occurred for 4 seconds at the beginning and 10 seconds at the end of the run. Across runs, the order of the video blocks was randomized. Video stimuli were presented at 512 x 512 px on a 41.5 x 41.5 cm screen, subtending approximately 9 x 9 degrees of visual angle in the participant's visual field. To ensure that participants remained alert throughout the experiment, they pressed a button whenever a red frame appeared around a video during one of the two video repetitions within a condition block. Such probes occurred 15 times per run and were counterbalanced so that each condition was probed once across all runs. All experimental protocols were presented using the Psychophysics Toolbox version 3 and MATLAB version R2016a.

Orthogonalizing the Feature Spaces. To facilitate our interpretation of each feature's contribution to the encoding models, the body parts and action target feature spaces were submitted to Principle Components Analysis (PCA), which extracts orthogonal components from the original feature space. Doing so enables weights to be fit over the body-part synergies rather than the body parts themselves – otherwise, with some kinds of regularized regression, highly correlated features (such as the thumb and index finger) might be assigned different and more variable weights, leading to the mistaken interpretation that a given region was tuned to the thumb but not the index finger.

However, it is important to note that this step is not necessary when using encoding modeling with ridge regularization, which handles issues of correlated feature predictors directly. In fact, doing so may hurt model prediction accuracy to some degree. We examined this possibility in the current data, and found that our step of orthogonalizing these predictors through PCA did not dramatically change the model's performance (average change in cross-validated r vs. a model fit on the original 25 features = 0.02, sd = 0.11 for set 1; average change = 0.01, sd = 0.11 for set 2). But, note that removing the PCA step may be a more optimal procedure for maximizing predictive accuracy in general.

PCA was performed separately on each feature space. The number of principle components (PCs) extracted was based on the number that cumulatively accounted for 95% of the variance in the feature ratings. This resulted in 7 body part PCs and 5 action target PCs (**Supplementary Figure 1**). The encoding modeling analysis was then performed over these 12 PC features. We followed a similar approach to fit a model based on the body parts and targets that were visible in the videos. These ratings were averaged across raters for each video, then binarized by rounding the average rating to either 0 or 1. PCA was then performed separately on visibility ratings for body parts and action targets. Based on the number of PCs that cumulatively accounted for 95% of the variance in the feature ratings, 11 body part visibility PCs and 4 target visibility PCs were extracted. The encoding modeling analysis was then performed over these 15 PC features.

Single-Subject Analyses. We also performed the encoding modeling and data-driven clustering analyses in each individual subject's data. Because subjects vary in the extent of their reliable coverage, making comparisons across subjects challenging, encoding modeling analyses were done in the same voxels as in the group (cross-sets reliability > 0.30 in the group data). Similarly, voxels were clustered into five networks using the voxels that were reliable and well-predicted ($r_{CV} > 0$ with fdr-corrected $q < 0.01$) in the group data. To make it possible to compare the clustering results across subjects, in **Supplementary Figure 4** the clusters are displayed on individual subjects' brains using a common colormap: clusters with a similar tuning profile across subjects are displayed in similar colors. This colormap was made by entering all subject's tuning profiles for all networks into a multi-dimensional scaling (MDS) analysis. The first three dimensions of the MDS solution were then extracted and used as R, G, and B values for each cluster's display color. Because some subjects did not have reliable neural responses in all of these regions, their results in **Supplementary Figure 2** and **Supplementary Figure 4** are ordered by the number of reliable voxels found in each subject's brain when reliable voxels were defined individually for each subject (cross-sets reliability > 0.30 in each subject's data).

Hierarchical Clustering. To validate our finding (based on k-means clustering) that the division between regions tuned to social and non-social features of actions emerges first, we also conducted a hierarchical clustering analysis. Voxels were grouped into a hierarchical tree based on the similarity of their feature weight profiles (the weights assigned by the voxel-wise encoding model to the 12 body-part and action-target features). This analysis was conducted separately over the data from our two video sets, using the group data. The analysis was restricted to the same set of voxels used in the k-means clustering analysis: voxels that were reliable across video sets and predicted well ($r_{CV} > 0$ with $q < 0.01$ after FDR-correction) by the model when it was fit to the data from either video set. Next, we used MATLAB's linkage function with the correlation distance metric to cluster voxels and then arrange them into a hierarchy based on the average distance between clusters. To determine the predominant division within this hierarchy, we examined the results at the point where the voxels branched into two clusters (**Supplementary Figure 6**). To determine the robustness of this clustering, we computed d-prime between the voxel groupings for video sets 1 and 2. To determine how similarly voxels were grouped by hierarchical clustering and k-means clustering, we computed d-prime between the voxel groupings for the two methods, within each video set.
Additional analyses revealed that subsequent divisions found by the hierarchical clustering analysis formed clusters that were very small (e.g., 30 voxels or fewer), and thus this supplemental analysis was not pursued further.

Models Based on Motion Features. The effects of low-level motion features were not investigated in-depth in this study. It is likely that motion plays an important role in action processing[2,3]. However, prior work has found that motion models such as motion energy[4] are only effective at capturing brain activity when subjects are required to maintain central fixation. When they view videos in a naturalistic fashion, moving their eyes around the frame as in the current study, this model breaks down unless neuroimaging is coupled with eye-tracking[4,5]. Further, our use of fMRI makes it challenging to collect measurements at the fine temporal scale at which motion features vary in our short video clips. Therefore, a combination of eye-tracking and fast fMRI sampling would be necessary to understand the how motion features contribute to the large-scale organization of action processing in the brain.

Relating Motion Span and Interaction Envelope. The span of motion present in each action video was measured in two ways. First, human raters (N = 182) completed an online experiment on Amazon Mechanical Turk, in which they watched each video and then answered the question, "How much movement does this action or activity involve for the average person?" using a 1 to 5 scale. Second, optical flow was calculated for every frame in the videos using the Horn-Schunck algorithm, implemented in MATLAB[6]. The proportion of pixels that contained movement (average optical flow magnitude > 0.01) was then calculated for each video. To relate these measures of movement to neural tuning patterns, we first obtained the overall neural response to each video for each of the five networks, averaging across all voxels in each network. Next, we calculated each network's motion sensitivity using a weighted average measure (i.e. for each network, the beta estimate for each video was multiplied by that video's motion span; these products were then summed together and divided by the total number of videos). Motion sensitivity is therefore an average response across all the videos, weighted by the amount of movement in each video. Each network's motion sensitivity was calculated separately for the two types of movement measurements (human ratings and optical flow; **Supplementary Figure 8**). This analysis was done separately for the two video sets.

Sociality and Transitivity Analysis. To compare our data to results reported in Wurm et al. (2017)[7], we searched for regions that were preferentially tuned to sociality (directed at a person) or transitivity (directed at an object). To do so, we fit a separate encoding model based on the raw, un-PC'd action target feature matrix. Then, in each voxel we compared the magnitude of the weight assigned to object targets with the weight assigned to person targets. Voxels were colored orange if the object weight was larger and pink if the person weight was larger. Saturation reflects the size of the difference between the weights (**Supplementary Figure 9**).

SUPPLEMENTARY REFERENCES

[1]Liu, T. T., Frank, L. R., Wong, E. C., & Buxton, R. B. Detection power, estimation efficiency, and predictability in event-related fMRI. *Neuroimage*, *13*(4), 759-773 (2001).

[2]Johansson, G. Visual perception of biological motion and a model for its analysis. *Perception & psychophysics*, *14*, 201-211 (1973).

[3]Isik, L., Tacchetti, A., & Poggio, T. A fast, invariant representation for human action in the visual system. *Journal of neurophysiology*, *119*, 631-640 (2017).

[4]Nishimoto, S., Vu, A. T., Naselaris, T., Benjamini, Y., Yu, B., & Gallant, J. L. Reconstructing visual experiences from brain activity evoked by natural movies. *Current Biology*, *21*, 1641-1646 (2011).

[5]Nishimoto, S., & Gallant, J. L. A three-dimensional spatiotemporal receptive field model explains responses of area MT neurons to naturalistic movies. *Journal of Neuroscience*, *31*, 14551-14564 (2011).

[6]Horn, B. K., & Schunck, B. G. Determining optical flow. *Artificial intelligence*, *17*, 185-203 (1981).

[7]Wurm, M. F., Caramazza, A., & Lingnau, A. Action categories in lateral occipitotemporal cortex are organized along sociality and transitivity. *Journal of Neuroscience*, *37*, 562-575 (2017).

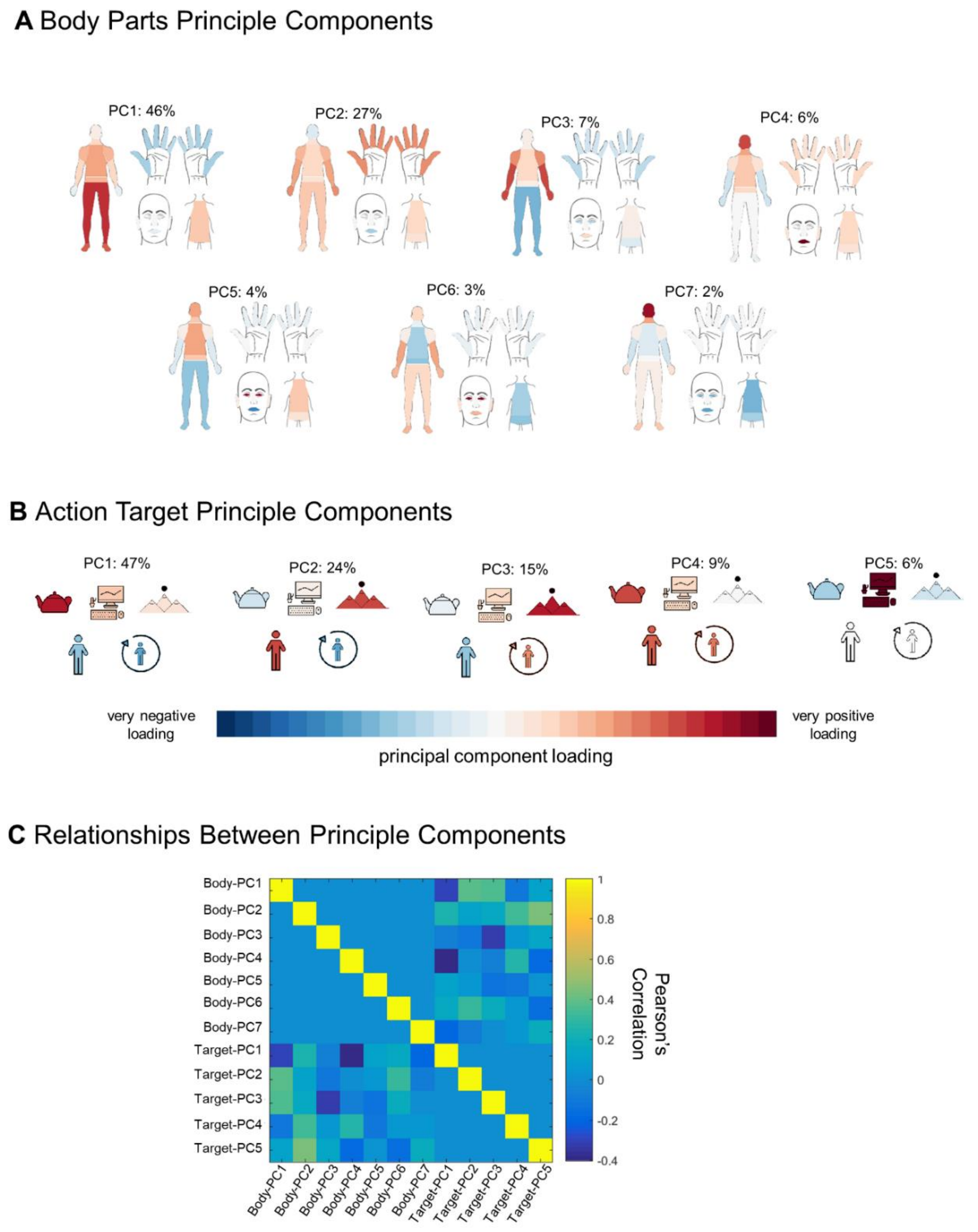

Supplementary Figure 1: Principle Components Analysis of Body Part and Action Target Feature Spaces. Visualization of the (A) body parts and (B) action target features after being reduced via Principle Components Analysis. Each feature (individual body part or action target) is colored according to the principle component's loading on that feature. Percentages indicate percent variance in the feature ratings explained by each component. Icons used to depict body part and target features were custom-made or based on images purchased from the Noun Project (Creative Commons License CC BY 3.0, https://creativecommons.org/licenses/by/3.0/), which were then colored and arranged by the authors. (C) Relationships between the Principle Components features, measured using Pearson's correlation.

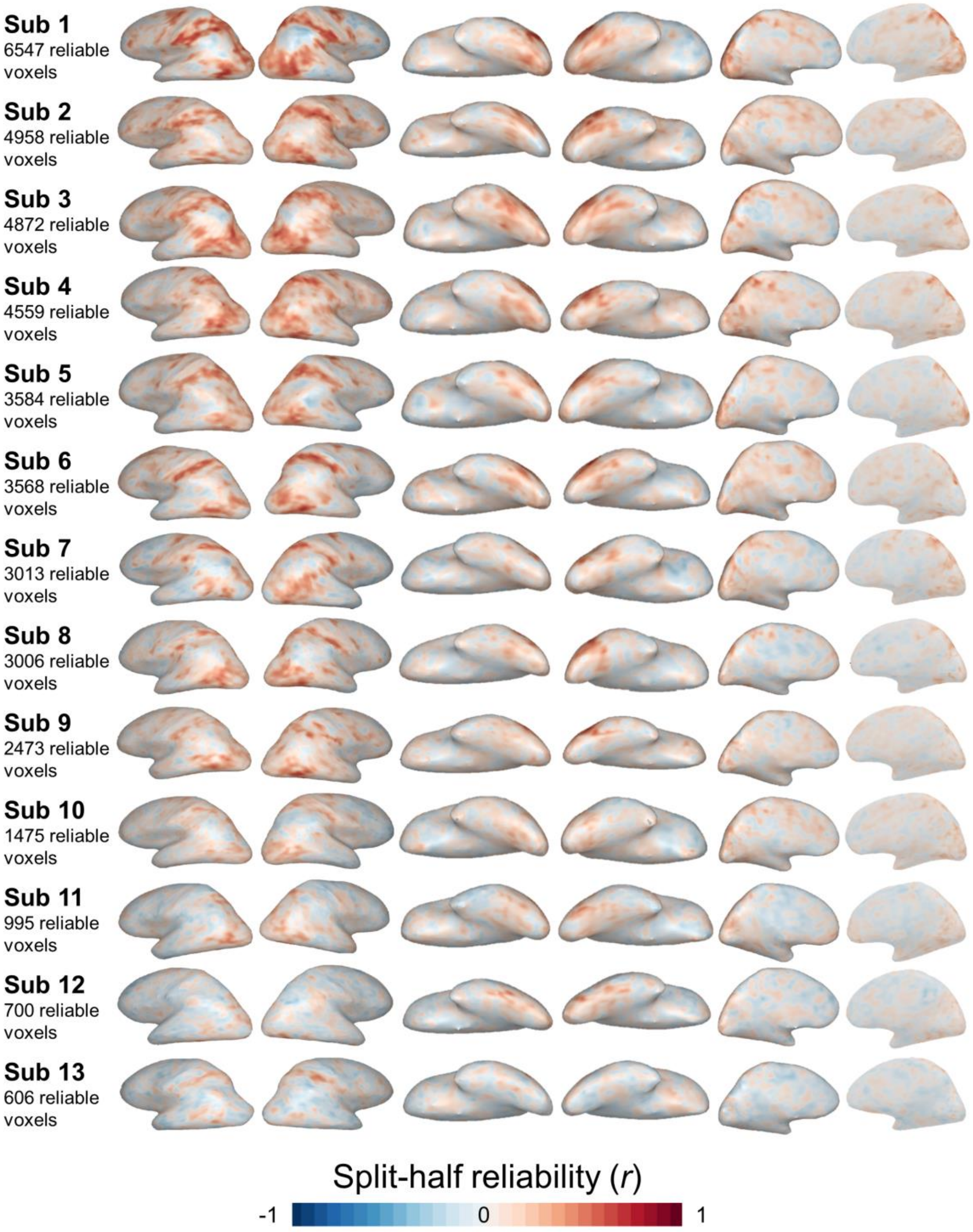

Supplementary Figure 2: Voxel-wise Reliability in Individual Subjects. Split-half reliability maps are shown for each subject. Subjects are ordered according to the number of voxels that survived the reliability-based inclusion threshold (split-half $r > 0.30$, which was an appropriate threshold for all subjects, as well as in the group data). All brain figures were created by the authors.

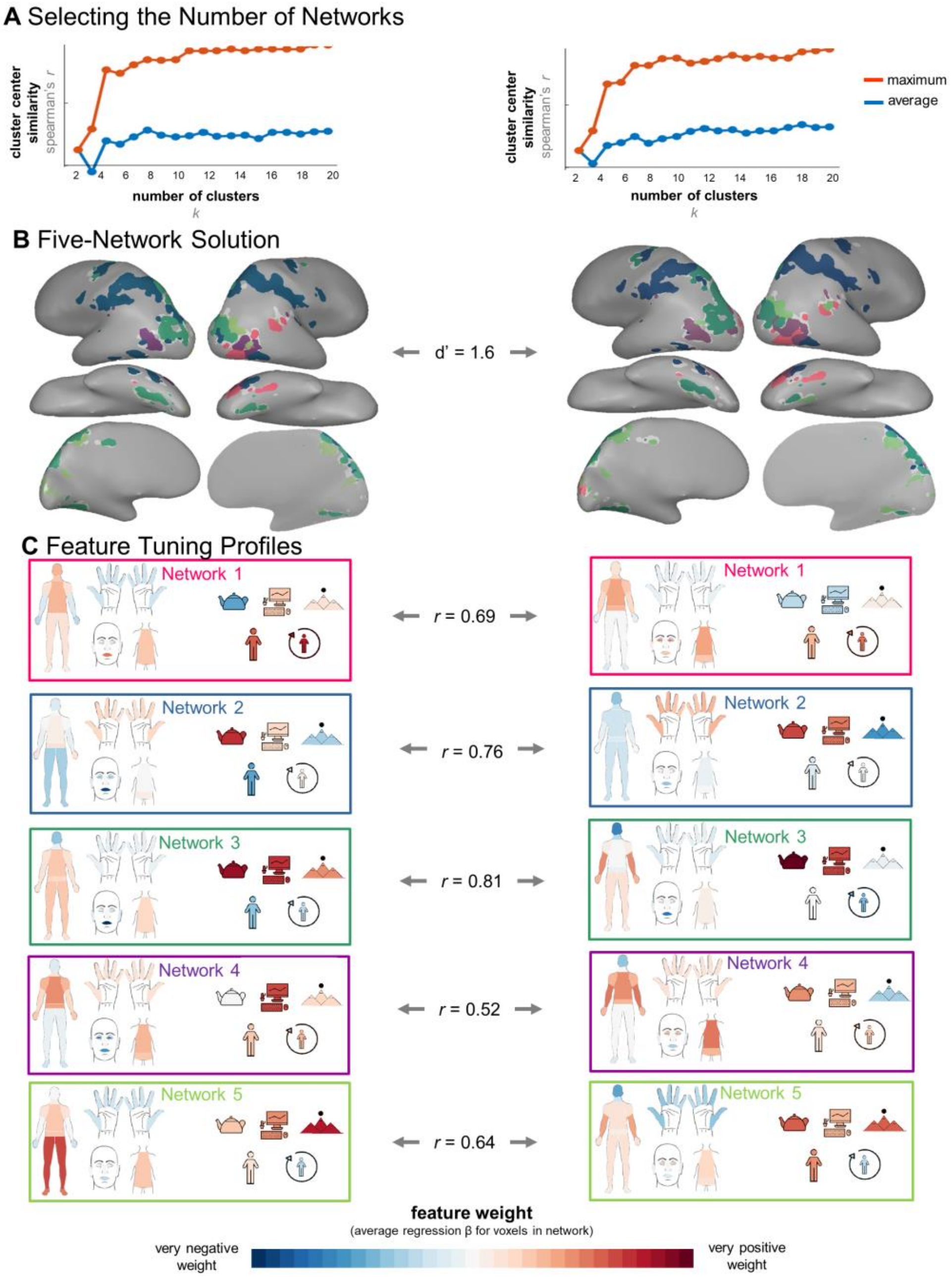

Supplementary Figure 3: Comparing 5-Network Solution Across Stimulus Sets. Results are shown for video set 1 (left) and set 2 (right). (A) K-means clustering was performed at every k from 2 to 20 (x-axis), and the resulting cluster centroid similarities (measured as the average (blue) and maximum (orange) correlation between the centers of every cluster) are plotted. (B) The 5-network structure is displayed for each video set. The match between the voxel assignments was computed using d-prime. (C) The feature tuning profile is shown for each cluster. Tuning profiles were compared across video sets using Pearson's correlation. Icons used to depict body part and target features were custom-made or based on images purchased from the Noun Project (Creative Commons License CC BY 3.0, https://creativecommons.org/licenses/by/3.0/), which were then colored and arranged by the authors. All brain figures were created by the authors.

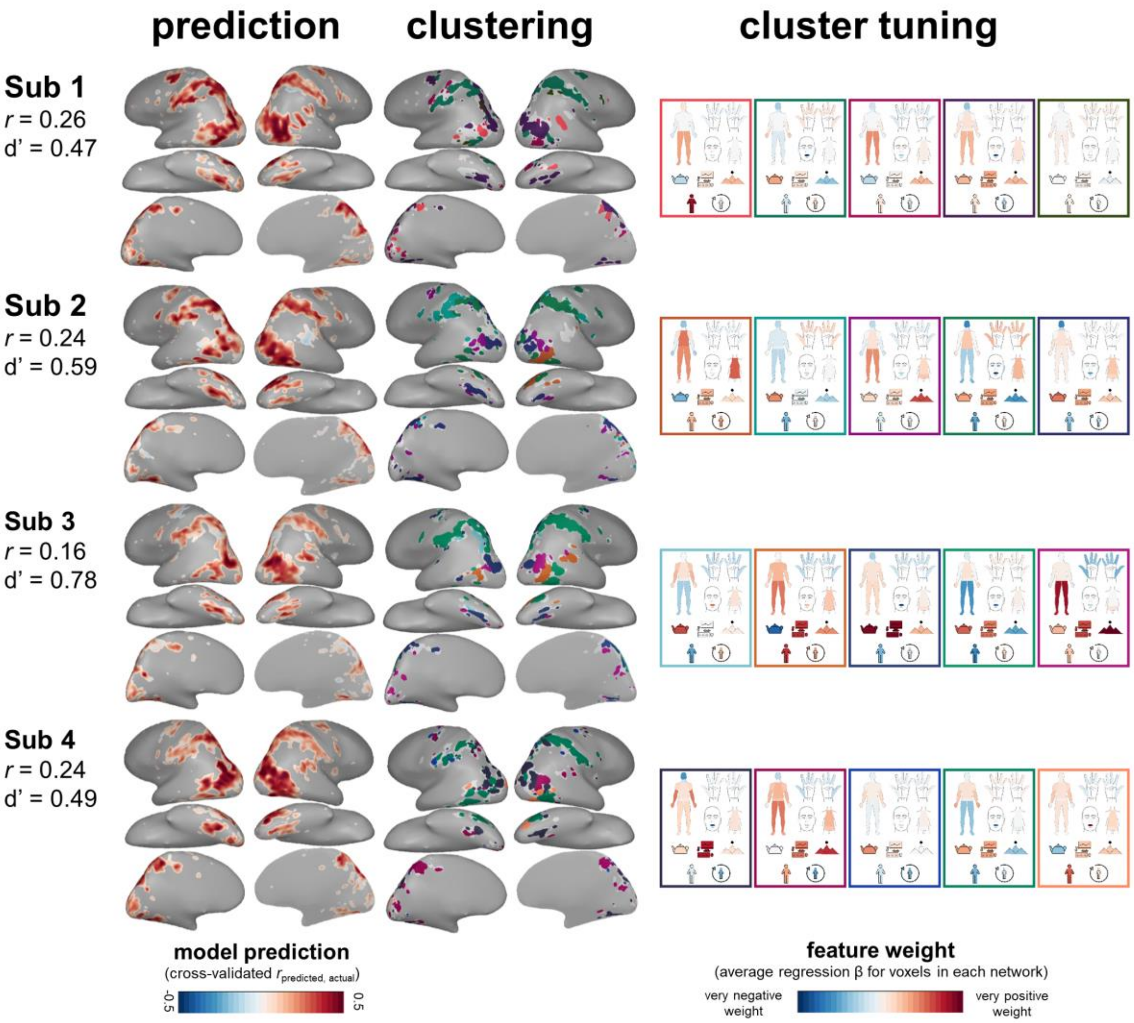

Supplementary Figure 4: Model Performance and Large-Scale Structure in Individual Subjects. Prediction performance for the action-target-body-part model and large-scale clustering of the model weights into five clusters are shown for each subject. For clustering results displayed on the brain, voxels are colored according to a common colormap: all voxels in a cluster are shown in the same color, and clusters with similar tuning profiles are shown in similar colors. In addition, each cluster's tuning profile is displayed to show which features the clusters are sensitive to. For each subject, we also list the median cross-validated prediction performance (*r*) and d-prime comparing how voxels were grouped in the single-subject and group data. All results are based on the data from video set 1. Subjects are ordered according to the number of voxels that survived the reliability threshold in their data (see **Supplementary Figure 2**). All single-subject analyses were conducted in the voxels used in the group analysis, though reliable coverage within these voxels differed across subjects. Figure continues for Subjects 5-13 on the next two pages. Icons used to depict body part and target features were custom-made or based on images purchased from the Noun Project (Creative Commons License CC BY 3.0, https://creativecommons.org/licenses/by/3.0/), which were then colored and arranged by the authors. All brain figures were created by the authors.

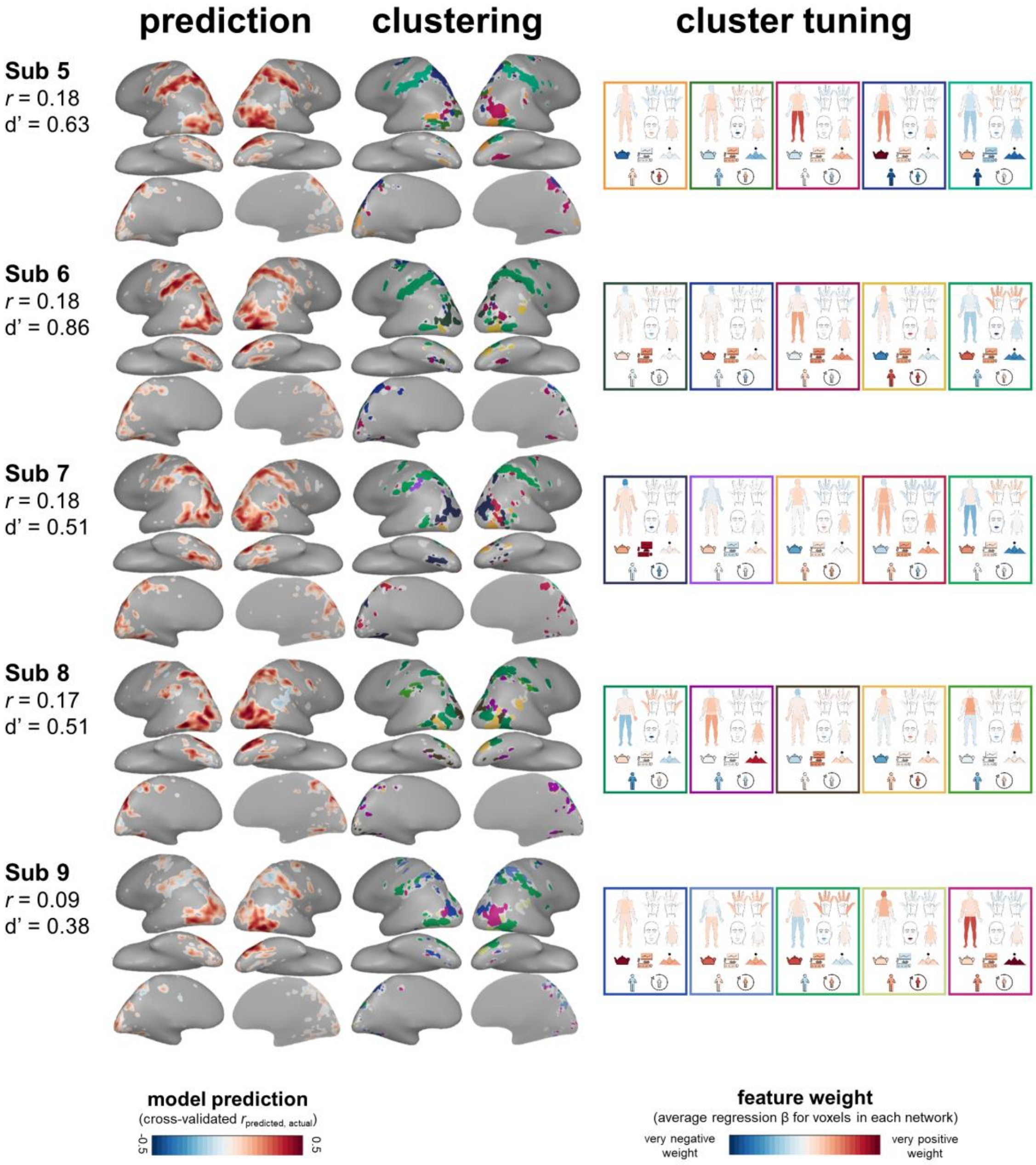

prediction
clustering
cluster tuning
Sub 5
r = 0.18
d' = 0.63
Sub 6
r = 0.18
d' = 0.86
Sub 7
r = 0.18
d' = 0.51
Sub 8
r = 0.17
d' = 0.51
Sub 9
r = 0.09
d' = 0.38
model prediction
(cross-validated r predicted, actual)
-0.5
0.5
feature weight
(average regression β for voxels in each network)
very negative weight
very positive weight

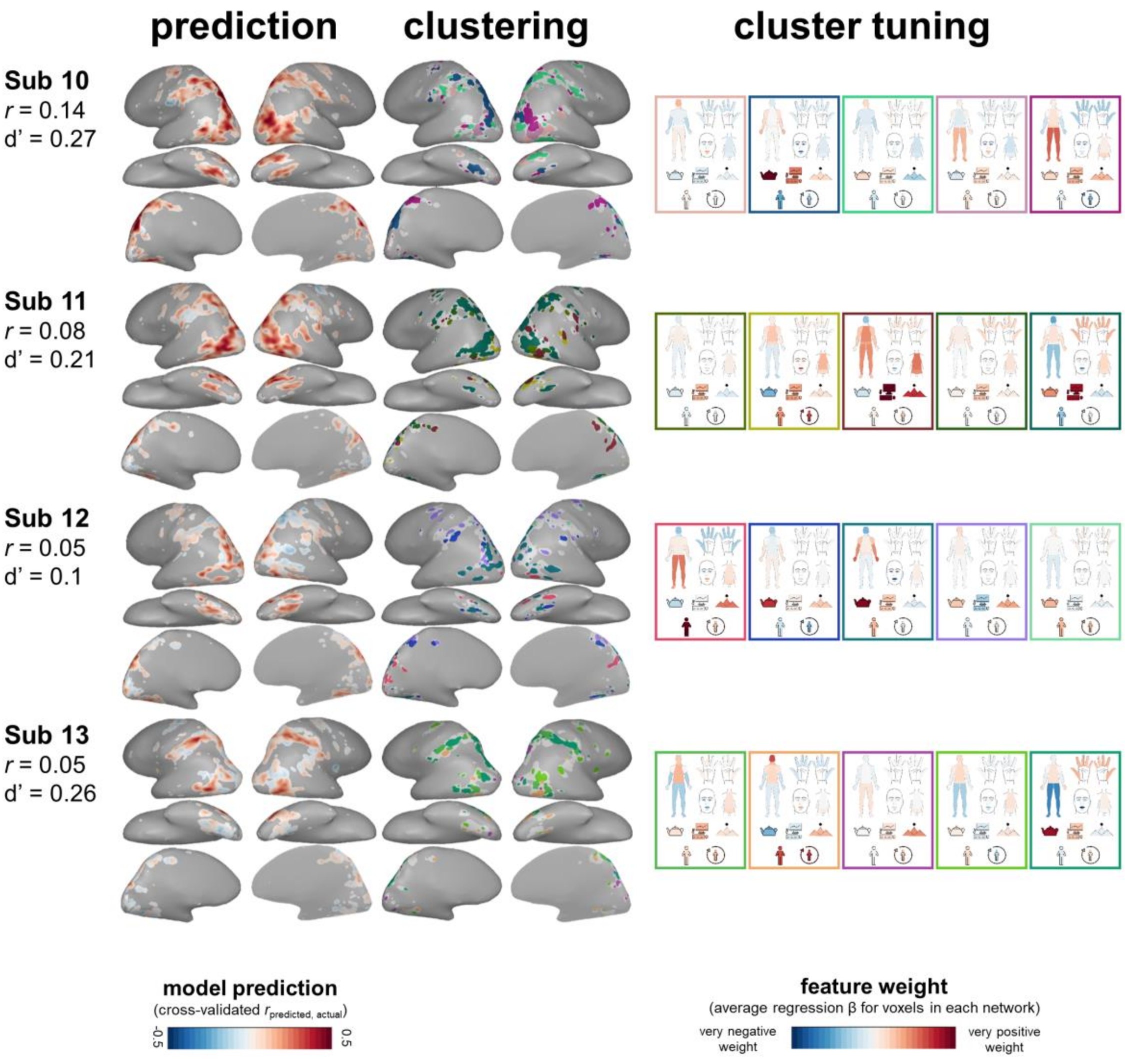
prediction
clustering
cluster tuning
Sub 10
r = 0.14
d' = 0.27
Sub 11
r = 0.08
d' = 0.21
Sub 12
r = 0.05
d' = 0.1
Sub 13
r = 0.05
d' = 0.26
model prediction
(cross-validated r predicted, actual)
-0.5
0.5
feature weight
(average regression β for voxels in each network)
very negative weight
very positive weight

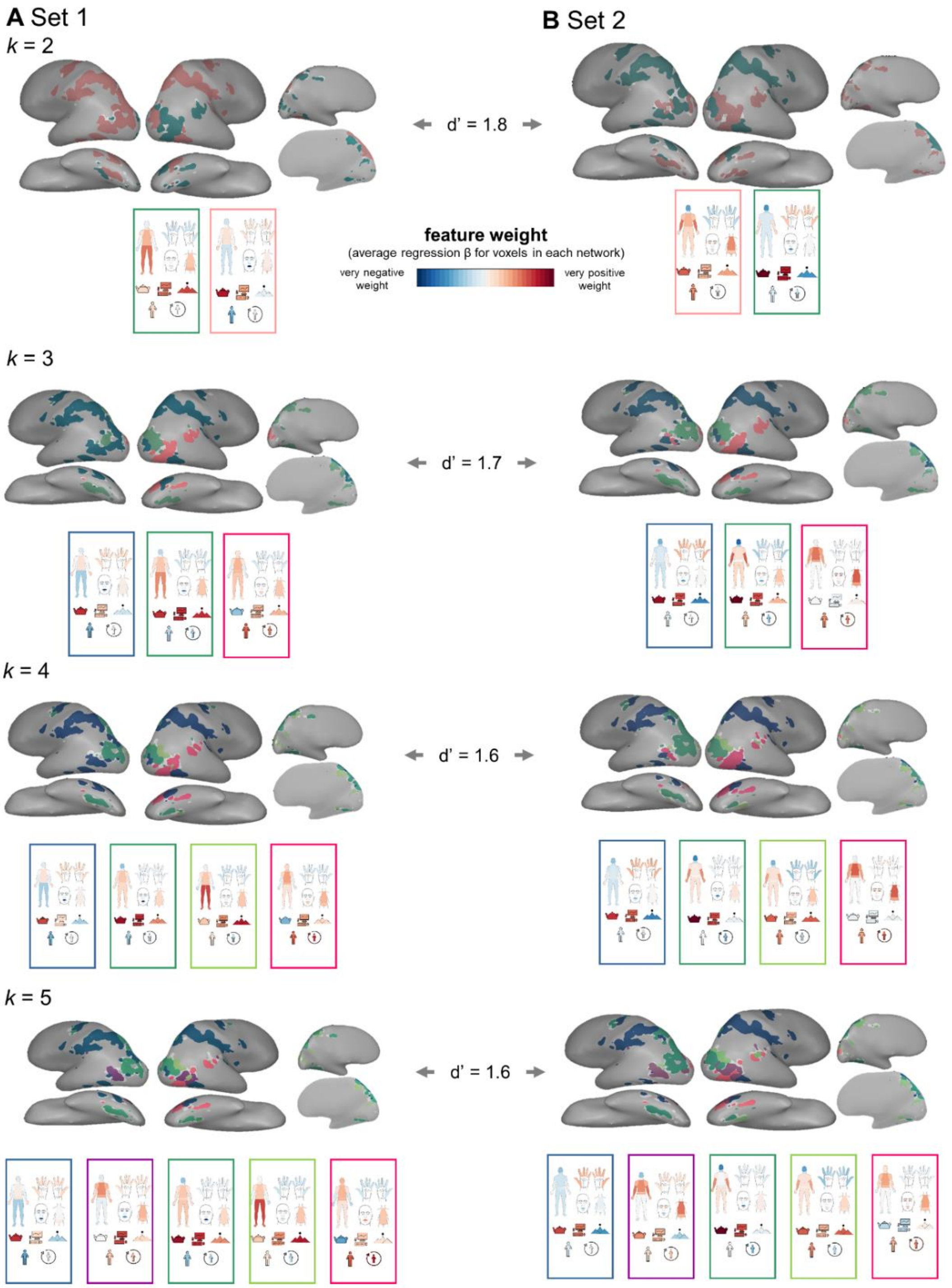

Supplementary Figure 5: The Emergence of Five Large-Scale Networks. Clustering solutions are shown at k = 2, 3, 4, and 5 clusters for (A) video set 1 and (B) video set 2. D-prime values indicate the correspondence between how voxels were clustered across the video sets. Icons used to depict body part and target features were custom-made or based on images purchased from the Noun Project (Creative Commons License CC BY 3.0, https://creativecommons.org/licenses/by/3.0/), which were then colored and arranged by the authors. All brain figures were created by the authors.

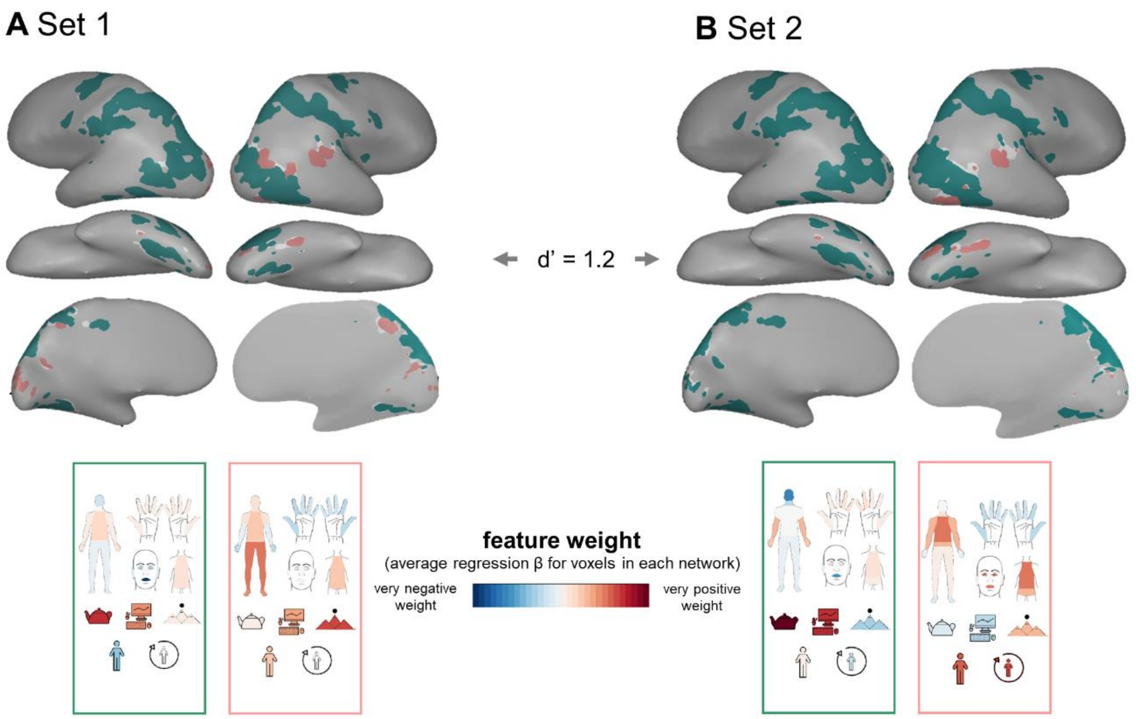

Supplementary Figure 6: Hierarchical Clustering of Voxel-wise Feature Tuning. Results of hierarchically clustering voxels into 2 clusters based on their feature tuning are shown for (A) video set 1 and (B) video set 2. The feature tuning profile (the average tuning across all voxels in the cluster) is shown for each cluster. D-prime value indicates the correspondence between how voxels were clustered across the video sets. The voxels were grouped similarly using this method compared to k-means clustering at k = 2 (**Supplementary Figure 5**; d-prime across clustering methods = 1.6 (set 1) & 0.6 (set 2)). Icons used to depict body part and target features were custom-made or based on images purchased from the Noun Project (Creative Commons License CC BY 3.0, https://creativecommons.org/licenses/by/3.0/), which were then colored and arranged by the authors. All brain figures were created by the authors.

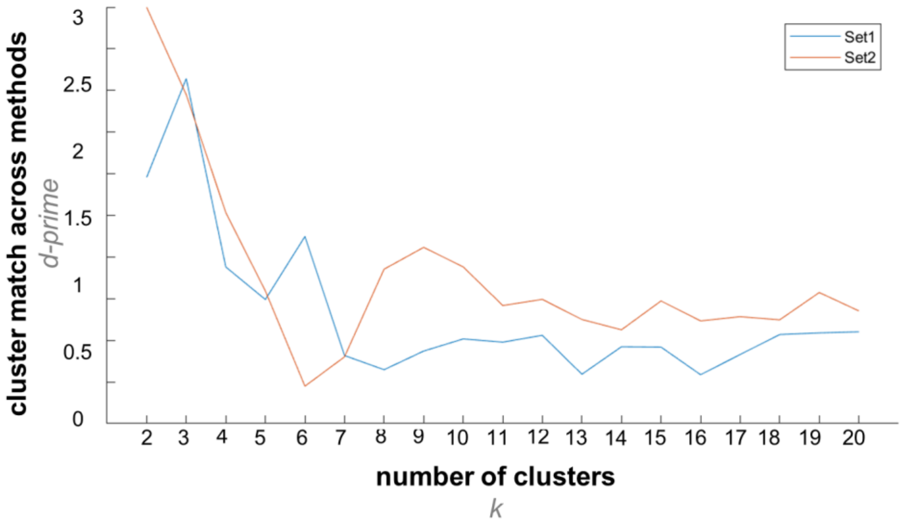

Supplementary Figure 7: Comparing Feature-Based and Feature-Free Clustering Analyses. Feature-free and feature-based clustering solutions were compared at a range of possible numbers of clusters (k-values) using d-prime. This was done separately for each video set.

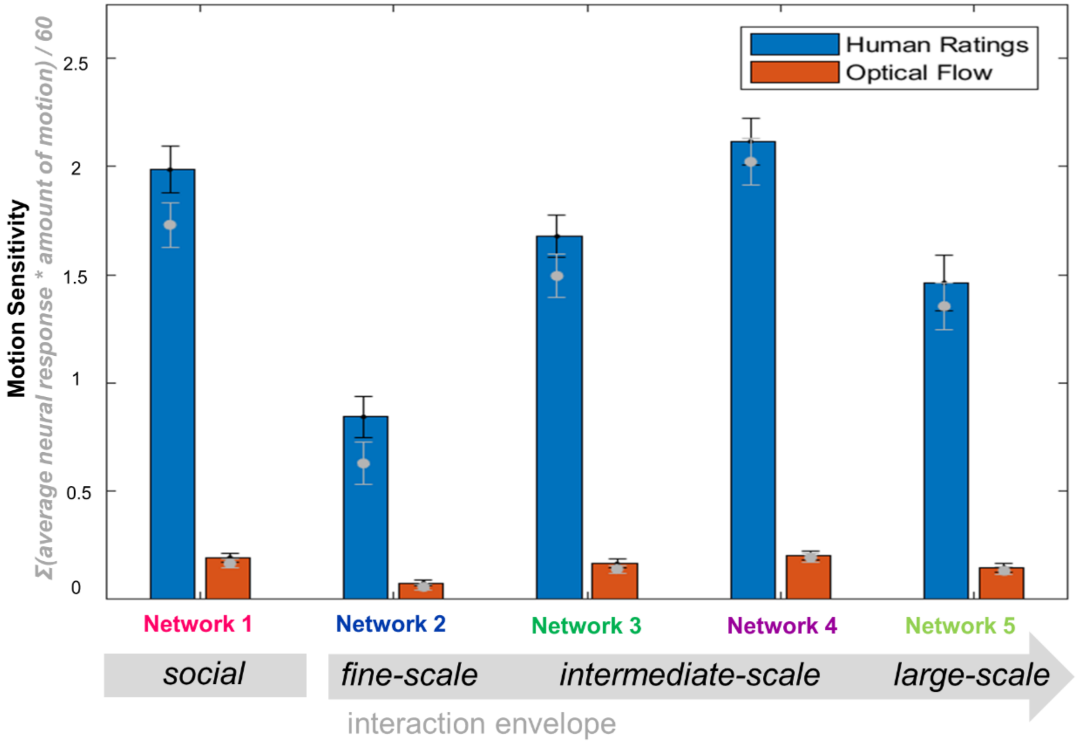

Supplementary Figure 8: Relating Interaction Envelope and Motion Span. Sensitivity to the span of motion in the action videos is plotted for the five action sub-networks. Motion Sensitivity was calculated as each network's average response over the action videos, weighted by the motion span apparent in each video (*N* = 60 videos). Blue bars depict motion sensitivity in video set 1 based on human ratings of how much the average actor moves to complete each action. Orange bars depict motion sensitivity in video set 1 based on the proportion of pixels in the video's frame containing movement during the course of the video, measured by optical flow. Error bars indicate the standard error of the mean, across 60 videos. Grey dots indicate the same values for video set 2 (*N* = 60 videos).

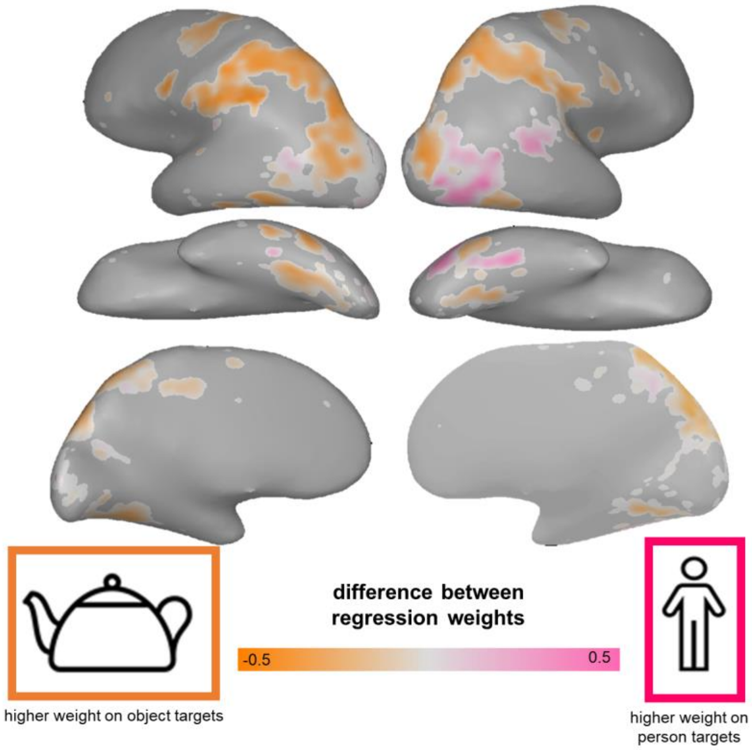

Supplementary Figure 9: Sociality and Transitivity (alternate feature spaces). Two-way preference map showing regions more related to person targets (“social” analog) or object targets (“transitive” analog). Voxels are colored according to the feature with the highest weight of the two possible targets: pink for person > object, orange for object > person. Color saturation reflects the strength of the voxel’s preference (person weight – object weight). Icons used to depict person and object targets were custom-made or based on images purchased from the Noun Project (Creative Commons License CC BY 3.0, https://creativecommons.org/licenses/by/3.0/), which were then colored and arranged by the authors. All brain figures were created by the authors.
