# Supplementary material for: Sociality and interaction envelope organize visual action representations
Source: Nat Commun. 2020 Jun 12;11:3002. doi: 10.1038/s41467-020-16846-w (PMC7293348; doi:10.1038/s41467-020-16846-w)
Supplement: Supplementary file 3 — Reporting Summary [file 41467_2020_16846_MOESM3_ESM.pdf]

nature research

Corresponding author(s): Leyla Tarhan

Last updated by author(s): Jul 11, 2019

Reporting Summary

Nature Research wishes to improve the reproducibility of the work that we publish. This form provides structure for consistency and transparency in reporting. For further information on Nature Research policies, see Authors & Referees and the Editorial Policy Checklist .

Statistics

For all statistical analyses, confirm that the following items are present in the figure legend, table legend, main text, or Methods section.

n/a	Confirmed	
☐	☒	The exact sample size (n) for each experimental group/condition, given as a discrete number and unit of measurement
☐	☒	A statement on whether measurements were taken from distinct samples or whether the same sample was measured repeatedly
☐	☒	The statistical test(s) used AND whether they are one- or two-sided<br>*Only common tests should be described solely by name; describe more complex techniques in the Methods section.*
☐	☒	A description of all covariates tested
☒	☐	A description of any assumptions or corrections, such as tests of normality and adjustment for multiple comparisons
☐	☒	A full description of the statistical parameters including central tendency (e.g. means) or other basic estimates (e.g. regression coefficient) AND variation (e.g. standard deviation) or associated estimates of uncertainty (e.g. confidence intervals)
☒	☐	For null hypothesis testing, the test statistic (e.g. F, t, r) with confidence intervals, effect sizes, degrees of freedom and P value noted<br>*Give P values as exact values whenever suitable.*
☒	☐	For Bayesian analysis, information on the choice of priors and Markov chain Monte Carlo settings
☐	☒	For hierarchical and complex designs, identification of the appropriate level for tests and full reporting of outcomes
☐	☒	Estimates of effect sizes (e.g. Cohen's d, Pearson's r), indicating how they were calculated

Our web collection on statistics for biologists contains articles on many of the points above.

Software and code

Policy information about availability of computer code

Data collection	Data were collected using custom code written in MATLAB version 2016a (fMRI experiment presentation), commercial Siemens software (used to collect raw MRI images), and custom code written in Javascript and HTML (Mechanical turk ratings experiments)
Data analysis	Data were analyzed using custom code written in MATLAB version 2017b, commercial software from BrainVoyager (version 2.8), and open-source Freesurfer software. MATLAB analysis code is available on the Open Science Framework (https://osf.io/uvbg7/)

For manuscripts utilizing custom algorithms or software that are central to the research but not yet described in published literature, software must be made available to editors/reviewers. We strongly encourage code deposition in a community repository (e.g. GitHub). See the Nature Research guidelines for submitting code & software for further information.

Data

Policy information about availability of data

All manuscripts must include a data availability statement. This statement should provide the following information, where applicable:

- Accession codes, unique identifiers, or web links for publicly available datasets
- A list of figures that have associated raw data
- A description of any restrictions on data availability

Group fMRI and ratings data, as well as code for the main analyses, are available at the Open Science Framework repository for this project (https://osf.io/uvbg7/, DOI: 10.17605/OSF.IO/UVBG7).

Field-specific reporting

Please select the one below that is the best fit for your research. If you are not sure, read the appropriate sections before making your selection.

Behavioural & social sciences study design

All studies must disclose on these points even when the disclosure is negative.

Study description	The data reported here are quantitative measurements of brain activity using functional MRI, as well as behavioral ratings.
Research sample	The research sample consisted of 13 members of the Harvard University community (5 males, 21-39 years). This sample is reasonably representative, especially since we did not predict that the visual system's organization would differ in another randomly-selected group of participants.
Sampling strategy	Participants were sampled based on convenience, taking into consideration their prior history of claustrophobia, ability to remain still during past fMRI experiments, and the absence of metal in their bodies. The sample size was selected based on common sample sizes used in the literature to investigate visual cortex representations; in addition, we performed a post-hoc analysis to investigate the reliability of these data across subjects, and found that it was very reliable within our chosen sub-set of voxels (average split-half correlation distance = 0.21, which was significantly lower than in scrambled data; $t(119) = 229.4$, $p < 0.001$). Therefore, we believe that this sample is reasonably representative of the human visual system, and not significantly influenced by outliers.
Data collection	Imaging data were collected using a 32-channel phased-array head coil with a 3T Siemens Prisma fMRI Scanner at the Harvard Center for Brain Sciences. In addition, human ratings data were collected using Amazon Mechanical Turk, where responses were in the form of clicks on a clickable map of the human body and multiple-choice responses to yes-or-no questions.
Timing	Data were collected between July, 2016 and December, 2016.
Data exclusions	No data were excluded from the analysis.
Non-participation	No participants dropped out of the study.
Randomization	Participants were not allocated into experimental groups.

Reporting for specific materials, systems and methods

We require information from authors about some types of materials, experimental systems and methods used in many studies. Here, indicate whether each material, system or method listed is relevant to your study. If you are not sure if a list item applies to your research, read the appropriate section before selecting a response.

Materials & experimental systems

n/a	Involved in the study	
x		Antibodies
x		Eukaryotic cell lines
x		Palaeontology
x		Animals and other organisms
	x	Human research participants
x		Clinical data

Methods

n/a	Involved in the study	
x		ChIP-seq
x		Flow cytometry
	x	MRI-based neuroimaging

Human research participants

Policy information about studies involving human research participants

Population characteristics	See above.
Recruitment	Participants were recruited from a registry of past fMRI participants and through word of mouth.
Ethics oversight	Harvard University Institutional Review Board.

Note that full information on the approval of the study protocol must also be provided in the manuscript.

Magnetic resonance imaging

Experimental design

Design type	The design of the study was "condition-rich," meaning that responses were collected during 5-second "mini-blocks", which are shorter than standard blocked designs but longer than standard event-related designed.
Design specifications	60 5-second blocks were included in each of 8 imaging runs. In addition, 4 15-second null blocks were interspersed throughout each run, with an additional 4 s. at the beginning and 10 s. at the end of each run.

Behavioral performance measures

To assess subjects' alertness throughout the experiment, we recorded their performance in a simple task (detecting the presence of a square red frame around the videos). Specifically, we recorded each button press and then assessed whether they detected the majority of the probe items (15 per run). Subjects were included as long as they missed fewer than 5 probe items.

Acquisition

Imaging type(s)

functional (8 runs) and structural (1 run)

Field strength

3T

Sequence & imaging parameters

Imaging data were collected using a 32-channel phased-array head coil with a 3T Siemens Prisma fMRI Scanner at the Harvard Center for Brain Sciences. High-resolution T1-weighted anatomical scans were acquired using a 3D MPRAGE protocol (176 sagittal slices; FoV = 256 mm; 1x1x1 mm voxel resolution; gap thickness = 0 mm; TR = 2530 ms; TE = 1.69 ms; flip angle = 7 degrees). Blood oxygenation level-dependent (BOLD) contrast functional scans were obtained using a gradient echo-planar T2* sequence (84 oblique axial slices acquired at a 25° angle off of the anterior commissure-posterior commissure line; FoV = 204 mm; 1.5x1.5x1.5 mm voxel resolution; gap thickness = 0 mm, TR = 2000 ms; TE = 30 ms; flip angle = 80 degrees; multi-band acceleration factor = 3).

Area of acquisition

High-resolution T1-weighted anatomical scans were acquired from the whole brain (176 sagittal slices FoV = 256 mm; 1x1x1 mm voxel resolution; gap thickness = 0 mm). Functional scans were acquired from a smaller region, which still included the whole brain (84 oblique axial slices acquired at a 25° angle off of the anterior commissure-posterior commissure line; FoV = 204 mm; 1.5x1.5x1.5 mm voxel resolution; gap thickness = 0 mm).

Diffusion MRI ☐ Used ☒ Not used

Preprocessing

Preprocessing software

Brain Voyager QX, version 2.8.4 was used to pre-process the anatomical and functional data. Functional preprocessing included slice scan-time correction, 3D motion correction, linear trend removal, temporal high-pass filtering (0.008 Hz cutoff), spatial smoothing (4 mm FWHM Kernel), and a transformation to Talairach coordinates. Whole-brain random-effect group GLMs were fit separately for each video set, as well as for both odd and even runs of each video set. In all cases, the design matrix included regressors for each condition block, specified as a square-wave regressor for each 5-second stimulus presentation time, convolved with a 2-gamma function that approximated the idealized hemodynamic response. Across these GLMs, the average variance inflation factor across conditions of the design matrix was 1.03 (where a value greater than 5 is considered problematic), and the average efficiency was 0.2 (Liu, Frank, Wong, & Buxton, 2001). Voxel time series were normalized within a run using a z-transform and corrected for temporal autocorrelations during GLM fitting. Beta weights extracted from these group-level random-effects GLMs were averaged across subjects for each voxel, and then taken as the primary measure of interest for all subsequent analyses. Each subject's cortical surface was reconstructed from the high-resolution T1-weighted anatomical scan using Freesurfer software, and one subject was selected as the display brain for the group data.

Normalization

Data were normalized to fit within Talairach coordinates. In addition, voxel time series from functional runs were normalized within a run using a z-transform and corrected for temporal autocorrelations during GLM fitting.

Normalization template

original Talairach

Noise and artifact removal

unctional preprocessing included slice scan-time correction, 3D motion correction, linear trend removal, temporal high-pass filtering (0.008 Hz cutoff), spatial smoothing (4 mm FWHM Kernel), and a transformation to Talairach coordinates.

Volume censoring

We did not perform volume censoring.

Statistical modeling & inference

Model type and settings

We used an encoding-model approach (Mitchell et al., 2008; Huth et al., 2012) to model each voxel's response magnitude for each action video as a weighted sum of the elements in the video's feature vector (e.g., individual body parts) using L2 ("ridge") regularized regression. The regularization coefficient (λ) in each voxel was selected for each voxel to minimize the mean-squared error of the fit in a 10-fold cross-validation procedure. Models were fit separately for the two video sets. To ensure that our models were not over-fit, we estimated their ability to predict out of sample using a leave-one-out cross-validation procedure. This was done by training the model iteratively on data from 59/60 videos in each voxel. We then calculated the predicted response magnitude for the held-out video (beta weights from the training model * feature vector for the held-out video). After 60 iterations, the predicted and actual data for the held-out actions were correlated to produce a single cross-validated r-value (rCV) for each voxel. All models were fit using responses from the group data. This procedure was performed separately using data from video set 1 and data video set 2.

Effect(s) tested

See above.

Specify type of analysis: ☐ Whole brain ☒ ROI-based ☐ Both

Split-half reliability was calculated for each voxel by correlating the betas extracted from odd and even runs of the main task. Reliability was calculated across sets by correlating odd and even betas from glms calculated over the two video sets. We then used a procedure from Tarhan & Konkle (under review,

Anatomical location(s)	https://osf.io/m9ykh/) to determine that any voxel with an average reliability of 0.3 or higher was a reasonable cutoff for inclusion in the feature modeling analysis. This cutoff held in both group and single-subject data; however, only voxels that were reliable at the group level were analyzed. Reliable voxels extensively covered the ventral and dorsal visual streams, as well as some portions of M1 and S1. However, they did not extensively cover early visual cortex, as this selection method requires responses to generalize over two different exemplars of the same action.
Statistic type for inference (See Eklund et al. 2016)	voxel-wise
Correction	no corrections were performed, as we conducted our analyses within a limited subset of reliably-responding voxels (see above).

Models & analysis

n/a	Involved in the study
☒	☐ Functional and/or effective connectivity
☒	☐ Graph analysis
☐	☒ Multivariate modeling or predictive analysis

Multivariate modeling and predictive analysis	See above.
